# Legal space for syringe exchange programs in hot spots of injection drug use-related crime

**DOI:** 10.1186/s12954-016-0104-3

**Published:** 2016-04-26

**Authors:** Sean T. Allen, Monica S. Ruiz, Jeff Jones, Monique M. Turner

**Affiliations:** Department of Epidemiology, Johns Hopkins University, 615 N. Wolfe St., Baltimore, MD 21205 USA; Department of Prevention & Community Health, Milken Institute School of Public Health at The George Washington University, 950 New Hampshire Ave, Suite 300, Washington, DC 20052 USA; Department of Health Policy & Management, Jiann-Ping Hsu College of Public Health at Georgia Southern University, PO Box 8015, Statesboro, GA 30460 USA

**Keywords:** Syringe exchange programs, Access, People who inject drugs, Harm reduction, Buffer zone policies

## Abstract

**Background:**

Copious evidence indicates that syringe exchange programs (SEPs) are effective structural interventions for HIV prevention among persons who inject drugs (PWID). The efficacy of SEPs in supporting the public health needs of PWID populations is partially dependent on their accessibility and consistent utilization among injectors. Research has shown that SEP access is an important predictor of PWID retention at SEPs, yet policies exist that may limit the geographic areas where SEP operations may legally occur. Since 2000 in the District of Columbia (DC), SEP operations have been subject to the 1000 Foot Rule (§48–1121), a policy that prohibits the distribution of “any needle or syringe for the hypodermic injection of any illegal drug in any area of the District of Columbia which is within 1000 feet of a public or private elementary or secondary school (including a public charter school).” The 1000 Foot Rule may impede SEP services in areas that are in urgent need for harm reduction services, such as locations where injections are happening in “real time” or where drugs are purchased or exchanged. We examined the effects of the 1000 Foot Rule on SEP operational space in injection drug use (IDU)-related crime (i.e., heroin possession or distribution) hot spots from 2000 to 2010.

**Methods:**

Data from the DC Metropolitan Police Department were used to identify IDU-related crime hot spots. School operation data were matched to a dataset that described the approximate physical property boundaries of land parcels. A 1000-ft buffer was applied to all school property boundaries. The overlap between the IDU-related crime hot spots and the school buffer zones was calculated by academic year.

**Results:**

When overlaying the land space associated with IDU-related crime hot spots on the maps of school boundaries per the 1000-ft buffer zone stipulation, we found that the majority of land space in these locations was ineligible for legal SEP operations. More specifically, the ineligible space in the identified hot spots in each academic year ranged from 51.93 to 88.29 % of the total hot spot area.

**Conclusions:**

The removal of the 1000 Foot Rule could significantly improve the public health of PWID via increased access to harm reduction services. Buffer zone policies that restrict SEP operational space negatively affect the provision of harm reduction services to PWID.

## Background

The USA is in the midst of an opioid abuse epidemic; according to the 2014 National Survey on Drug Use and Health (NSDUH), there are an estimated 4.3 million persons who engaged in non-medical use of prescription painkillers in the last month [1]. This statistic serves as a call to action as persons who misuse prescription opioids may transition to heroin use [2] and experience increased vulnerability for a number of blood-borne infections. While this epidemic is not new, it is one that has seen significant expansion to include a greater diversity of affected populations (e.g., non-minority, rural, and suburban populations). The recent outbreaks of HIV that were linked to the injection of prescription opioids in rural locations, such as in Indiana [3], demonstrate a need not only for expanded mental health and addiction treatment services but also for more comprehensive approaches to both preventing substance abuse and reducing harm for existing persons who inject drugs (PWID).

While substance use cessation is the ultimate goal of drug treatment programs, total sobriety is a difficult state to achieve and maintain. For example, a 12-year longitudinal study of substance users engaged in drug treatment programs found that 14.3 % had experienced a single relapse and 36.9 % had experienced multiple relapses [4] from sobriety attempts. In light of the complicated nature of addiction and the difficulty of accessing treatment on demand, harm reduction services become even more critical for maintaining health among PWID. While there is no universal definition for harm reduction, the Harm Reduction Coalition states that “Harm reduction is a set of practical strategies and ideas aimed at reducing negative consequences associated with drug use. Harm Reduction is also a movement for social justice built on a belief in, and respect for, the rights of people who use drugs.” [5]. The need for expansion of harm reduction services is particularly true in places such as the District of Columbia (DC), which has epidemic rates of both HIV and HCV. While incidence of HIV among PWID in DC has decreased, injection drug use remains the third leading cause of HIV transmission [6]. Additionally, DC-specific data from the 2010 National Health and Behavior Survey (NHBS) showed that 90 % of PWID surveyed reported being HCV positive [7].

Harm reduction interventions—such as syringe exchange programs (SEPs)—play a critical role in reducing the spread of HIV and HCV among PWID. There is copious evidence that SEPs are an effective and cost-effective public health strategy for the prevention of HIV and HCV infections among PWID via provision of sterile injection equipment and reducing risky injection behaviors [8–12]. Notably, a 2015 study in DC found that, following a policy change allowing for the municipal funding and expansion of syringe access services, an estimated 120 HIV infections were averted among PWID; these averted infections resulted in the saving of approximately 45.6 million USD that would have been spent for lifetime treatment of these HIV infections [12]. In addition, SEPs may also provide public health benefits for PWID via education about overdose prevention (e.g., administration of naloxone) or providing referrals to medical and social services (e.g., health screenings) [13, 14]. Moreover, the incorporation of harm reduction strategies into public health interventions for PWID populations may help resolve other unmet health care access needs for this population [15].

Structural factors—i.e., social, environmental, and policy factors that are outside the control of the individual—may disproportionately affect PWID access to harm reduction services. Research has empirically demonstrated the importance of access to harm reduction services among PWID populations; for example, Williams et al. found that distance to SEP sites affect injection practices differentially by race and that Latinos’ injection behaviors were more distance-dependent than other races in that the odds of sharing injection equipment increased as the number of miles from SEP services increased [16]. Other research has found that PWID who reside more than a mile from a SEP are more likely to have injected with a used syringe in the prior 6 months [17]. These data are especially concerning for DC PWID, a population that, on average, travels approximately 2.75 miles from a home residence to SEPs [18].

Other structural factors influencing access to SEP services are the various types of legislation that limit implementation of or access to SEP services. At the Federal level, the use of Federal monies to support SEPs was prohibited in its entirety [19, 20]. This policy was changed at the end of 2015 to allow the use of Federal funds for SEP operations (e.g., personnel), but funds still cannot be used by programs to purchase sterile injection equipment. At the state and local level, drug paraphernalia laws may outlaw the possession of injection equipment [14]. The enforcement of drug policies may impose restrictions and/or barriers for SEP operations and result in negative health consequences for PWID; for example, research has documented that PWID are sensitive to police activity and that concern about arrest may lead persons to not seek out and/or carry sterile syringes [21–23]. In jurisdictions where law enforcement implement targeted enforcement campaigns, such as in areas where drugs are bought/sold, PWID may have insufficient sterile syringe coverage due to fears of criminalization. The negative effects of insufficient syringe access may also be intensified by co-occurring policies that further limit access to sterile injection equipment.

Buffer zone policies are examples of policy-level impediments to syringe access and SEP implementation. These policies limit where SEPs can legally operate based on geography (e.g., proximity to schools, day cares, or public spaces). For example, a buffer zone policy in Pittsburgh, PA, banned SEP operations within 1500 feet of schools [24]. A comparable policy in Denver, CO, also restricted SEP operations from within 1000 feet of a school or day care center [25].

In DC, SEPs must abide by the 1000 Foot Rule (§48–1121), a policy that, since 2000, has prohibited the distribution of “any needle or syringe for the hypodermic injection of any illegal drug in any area of the District of Columbia which is within 1000 feet of a public or private elementary or secondary school (including a public charter school)” [26]. With SEP efficacy dependent on injectors having access to services, it is important to understand the effect of the 1000 Foot Rule on the amount of legal SEP operational space in areas with high concentrations of injection drug use (IDU)-related activities. The 1000 Foot Rule may impede SEP services in areas where injections are happening in “real time”—such as the locations where drugs are purchased, exchanged, and used—and, therefore, where there is an even more urgent need for harm reduction services.

No research has examined the effect of the 1000 Foot Rule on the amount of legal SEP operational space in areas where IDU-related activities are highly concentrated. The purpose of this research is to extend our understanding of the impact of the 1000 Foot Rule by examining the effect of this policy (from its implementation in 2000 to 2010) on legal SEP operational in areas with disproportionate concentrations, or hot spots, of IDU-related crime (e.g., heroin possession/distribution) in DC. We hypothesized that the majority of the land space in the IDU-related crime hot spots would be ineligible for SEP operations due to the 1000 Foot Rule in all years since its implementation.

## Methods

### School buffer zones

The methods used to evaluate the effects of the 1000 Foot Rule on the overall amount of SEP operational space can be found in related research [27, 28]. For brevity, a reduced version of these methods is provided here. School operations data were accessed via publicly available sources (e.g., annual reports, school directories), online searches, and Freedom of Information Act (FOIA) requests to the DC Public Charter School Board and DC Public Schools. Data were also abstracted from publicly available resources and datasets created by the National Center for Education Statistics, the DC Office of the Chief Technology Officer (OCTO), http://www.education.com, and the National Association of Independent Schools [29–32]. These sources were used to ascertain the address of the schools, what grade(s) were taught during each school year, and what year(s) the schools were in operation.

The DC Master Address Repository (MAR) Geocoder, a publicly accessible tool that allows the user to search a database of addresses, blocks, intersections, place names, and other location identifiers in the District, was used to download data about each school [33]. These data included the Square Suffix Lot (SSL) identifier for each location. The SSL identifier is used by the DC Government for city planning processes and taxation assessments. A dataset of approximate land parcel boundaries (including SSL data) was downloaded from the DC GIS Data Clearinghouse [34]. The output from the DC MAR application was then matched to the school property dataset using the SSL identifier. ArcMap v10.2.1 was used to extract the approximate property boundaries of each school.

The school operations data were then grouped by academic year. The academic year was used to frame the analyses because of its direct impact on the application of the 1000 Foot Rule. More specifically, the policy implications are dependent on school operational years (i.e., when schools are in session) rather than fiscal or calendar years. We defined the academic year as September to May. This decision was based on the fact that the majority of DC schools defined their academic years as extending from late August or early September to May. Efforts were undertaken to classify the June, July, and August months accurately based on how schools treated these months in relation to their conceptualization of the academic year/school operations, but the schools were heterogeneous in terms of how the summer months related to academic year designation (e.g., some schools were in session year round while others operated on semester, trimester, or quarter systems).

A 1000-ft buffer was then applied to the school property boundaries and to the point location of those schools that did not generate a match to DC MAR data. School buffers were combined into a single continuous layer via the Merge and Dissolve tools in ArcMap. All areas where SEP operations could not occur due to restrictions other than the 1000 Foot Rule (e.g., areas under Federal jurisdiction, such as national parklands and military installations) were also mapped using ArcMap by academic year. Bodies of water were also included in the analyses as they pose obvious geographic impediments to service delivery.

### IDU-related crime hot spots

After the 1000-ft buffers were applied to the school property boundaries, analyses were conducted to determine the amount of land space ineligible for SEP operations in IDU-related crime hot spots. These locations were determined by conducting optimized hot spot analyses of publicly available data from the DC Metropolitan Police Department (DC MPD) that detailed all of the charges that occurred in the District from 2000 to 2011 (*n* = 778,149). The DC MPD data included charge descriptions, latitude and longitude coordinates of where the charge occurred, and the time/date of when the charge occurred.

Only charges involving heroin distribution or possession were used in the identification of IDU-related crime hot spots. Rationale for this decision stemmed from the majority of PWID who accessed syringe exchange services in the DC metropolitan area most frequently reporting injecting heroin and that heroin is their drug of choice [35]. Drug paraphernalia charges were also excluded from the hot spot analyses because the MPD dataset did not provide sufficient information in the charge descriptions to determine if the charge was IDU-related. All drug paraphernalia charges were reported as “UCSA [United States Controlled Substances Act] Possession of Drug Paraphernalia” violations, but with no other information that could be used to differentiate probable injection or non-injection drug use. It was also possible for individuals to be charged with multiple infractions at a single event. To that end, the total number of charges could be greater than the total number of charge events counted in a given time period.

The heroin-related charges were divided into corresponding academic years and analyzed for the presence of hot spots via the optimized hot spot analysis tool in ArcMap. Only those hot spots that were significant at the *p* < .05 level were included in the analyses. After conducting the hot spot analysis for each year of interest, the identified hot spots were aggregated into a single continuous layer (by academic year) using the Dissolve tool in ArcMap. The Clip and Calculate geometry tools were used to determine the square mileage of the hot spots in DC overall. These tools were also used to calculate the overlap between the hot spots and the school buffer zones. The resulting data were used to calculate the percent impact of the 1000 Foot Rule on the amount of legal SEP operational space in the identified hot spots of IDU-related crime (i.e., the percent of the total area of the hot spots that fell within 1000 feet of a school).

## Results

The DC MPD data contained records for 778,149 charges that occurred from 2000 to 2011. These charges were distributed among 565,345 unique charge events. There were 12,965 IDU-related (heroin possession or distribution) charges that were distributed among 11,578 unique charge events. Location data were available for 96.5 % (*n* = 11,168) of these unique heroin-related charges. After dividing the data into academic years and excluding those charges that occurred during the June, July, and August months, 74.5 % (*n* = 8320) of the charges remained. These data formed the basis for the hot spot analyses. The number of IDU-related charges per academic year ranged from 558 to 863 (Table 1).

**Table 1:** IDU-related charges by academic year

Academic year	Total number of IDU-related charges by academic year
2000	702
2001	762
2002	784
2003	863
2004	790
2005	777
2006	558
2007	745
2008	764
2009	738
2010	598

**Fig. 1:**
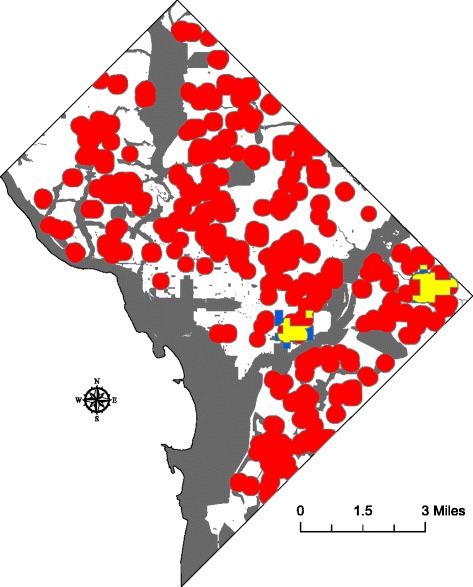
IDU-related crime hot spots and school buffer zones in the 2008 academic year

The optimized hot spot analyses showed that IDU-related crime hot spots were not present in all years of the study; no hot spots were identified in the 2001, 2003, and 2005 academic years. In those years in which hot spots were identified, their total square mileage ranged from 0.222 to 1.018 square miles. Analyses of the overlap between the hot spots of IDU-related crime and the school buffer zones showed that the majority of the land space in the hot spots fell within the school buffers. These data supported our hypothesis; notably, the amount of overlap ranged from 51.93 to 88.29 % over the study period. These data are summarized in Table 2. Figure 1 is an exemplar image of these findings for the 2008 academic year.

**Table 2:** IDU-related crime hot spots by academic year

Academic year	Total square mileage of IDU-related crime hot spots	Total square mileage of overlap between IDU-related crime hot spots and school buffer zones	Percent of overlap between IDU-related crime hot spots and school buffer zones
2000	0.462	0.313	67.75
2001	0.000	0.000	0.00
2002	0.521	0.377	72.36
2003	0.000	0.000	0.00
2004	0.493	0.256	51.93
2005	0.000	0.000	0.00
2006	0.624	0.541	86.70
2007	0.699	0.599	85.69
2008	1.018	0.823	80.84
2009	0.366	0.217	59.29
2010	0.222	0.196	88.29

## Discussion

This research demonstrates that buffer zone policies may impose significant barriers on SEP access within areas that have high concentrations of IDU-related crime. We hypothesized that the majority of the square mileage of land space in the IDU-related crime hot spots would be ineligible for SEP operations. The data supported our hypothesis by showing that, in years in which hot spots of IDU-related crime were identified, the majority of their area fell within the school buffer zones. While the total square mileage of the hot spots in each year was relatively small, it remains notable that more than 50 % of their total area fell within the school buffers each year.

PWID face marginalization, stigmatization, and other structural barriers that influence their health; the 1000 Foot Rule and other comparable buffer zone policies have the potential to exacerbate the negative health outcomes among injectors by limiting their access to services that may help resolve unmet needs. The removal of these policies could dramatically increase PWID access to harm reduction services in risk environments (e.g., hot spots of IDU-related crime); such services are not only critical to positively reinforce sterile syringe usage and not sharing injection equipment but are also critical for overdose education and prevention. Future work should examine how the removal of buffer zone policies affects the public health of PWID and members of their social, sexual, and drug-using networks.

Though the 1000 Foot Rule has been in place since 2000, the number of new HIV infections attributed to IDU in the District has declined substantially over time. In 2008 and 2012, there were 109 and 21 new HIV infections, respectively, attributed to IDU [6]. This decline is most likely reflective of the expansion of SEP services in the District. It is also possible that the identified hot spots of IDU-related crime are not areas that are most salient in HIV acquisition (i.e., SEP services are being delivered in risk environments for HIV acquisition that are outside the hot spot areas). Future work should explore how SEP services are being delivered in the District and how SEP service providers and their clients are navigating the myriad factors—including the 1000 Foot Rule—that affect SEP access and utilization.

These findings have a number of limitations, a significant one being the lack of available data pertaining to adherence to the 1000 Foot Rule by harm reduction providers and enforcement of the 1000 Foot Rule by law enforcement. SEP providers are obligated to keep track of the ever-changing landscape of schools in operation and how the 1000 Foot Rule applies to these property boundaries. Given how quickly schools of various sorts (e.g., private, charter) open in urban areas, keeping track of such information poses a significant struggle. To that end, providers may be simply using their best judgment in determining where SEP service delivery is legal.

An additional limitation of this research pertains to the utilization of DC MPD crime data. Only charges that indicated heroin possession or distribution were included in the hot spot mapping. It is possible that other drug charges (e.g., possession of cocaine, amphetamines) could have indicated possible injection drug use. Another limitation related to the DC MPD data is that we cannot ascertain whether the identified hot spots are true areas of need for harm reduction services (meaning, places where people both buy and inject their drugs) or if they are merely areas of targeted enforcement.

This research had multiple strengths. Foremost, DC MPD data that spanned more than a decade were used to map the locations of hot spots for IDU-related crime. No other study has examined data of this type over as long of a study period. Further, no other studies have quantified the square mileage of the hot spots of IDU-related crime in DC, nor has any research been conducted to quantify the overlap of the IDU-related crime hot spots with school buffers. This is a particular strength of this study because previous research has suggested that work should be undertaken to locate places of importance for PWID to inform the placement and accessibility of HIV and overdose prevention programs [36]. Our research not only locates possible areas of importance for HIV prevention services for PWID but also places them in the context of the 1000 Foot Rule.

## Conclusions

In conclusion, the results of this research demonstrate the magnitude of the effects of the 1000 Foot Rule on legal SEP operational space in areas with high concentrations of IDU-related crime. The majority of land space in the IDU-related crime hot spots was ineligible for legal SEP operations, and, as a result, may negatively affect the access and utilization of harm reduction services among injectors. The removal of the 1000 Foot Rule could improve the public health of PWID by increasing access to harm reduction services where drug use is happening in real time.

## Abbreviations

DC: Washington, DC
PWID: persons who inject drugs
SEPs: syringe exchange programs
